# Resistance Mechanisms and Molecular Docking Studies of Four Novel QoI Fungicides in *Peronophythora litchii*

**DOI:** 10.1038/srep17466

**Published:** 2015-12-14

**Authors:** Yuxin Zhou, Lei Chen, Jian Hu, Hongxia Duan, Dong Lin, Pengfei Liu, Qingxiao Meng, Bin Li, Naiguo Si, Changling Liu, Xili Liu

**Affiliations:** 1 Department of Plant Pathology, College of Agriculture and Biotechnology, China Agricultural University, Beijing, 100193, China; 2 College of Forestry, Beijing Forestry University, Beijing, 100083, China; 3 College of Agro-grassland Science, Nanjing Agricultural University, Nanjing, 210095, China; 4 Department of Chemistry, College of Science, China Agricultural University, Beijing, 100193, China; 5 State Key Laboratory of the Discovery and Development of Novel Pesticide, Shenyang Research Institute of Chemical Industry co., Ltd., Shenyang, 110021, China

## Abstract

*Peronophythora litchii* is the causal agent of litchi downy blight. Enestroburin, SYP-1620, SYP-2815 and ZJ0712 are four novel QoI fungicides developed by China. Eight mutants of *P. litchii* resistant to these QoI fungicides and azoxystrobin (as a known QoI fungicide) were obtained in our preliminary work. In this study, the full length of the cytochrome b gene in *P. litchii*, which has a full length of 382 amino acids, was cloned from both sensitive isolates and resistant mutants, and single-site mutations G142A, G142S, Y131C, or F128S were found in resistant mutants. Molecular docking was used to predict how the mutations alter the binding of the five QoI fungicides to the Qo-binding pockets. The results have increased our understanding of QoI fungicide-resistance mechanisms and may help in the development of more potent inhibitors against plant diseases in the fields.

*Peronophythora litchii*, the causal agent of litchi downy blight, is a transitional species between *Phytopthora* and *Peronospora*^1^,^2^,^3^. This oomycete was first described in Taiwan^4^ and can reduce litchi yield and quality by attacking panicles, new shoots, leaves, and young and mature fruit^5^,^6^, causing severe pre-harvest and post-harvest disease of litchi. With the increase of the popular frequency and prevalence of this disease, it has become an important barrier of litchi production. Efficient control of litchi downy blight is mainly based on application of agrochemicals including traditional fungicides, such as mancozeb, cymoxanil, and metalaxyl, and the recently registered QoI fungicide azoxystrobin^5^,^7^.

QoI fungicides, which interfere with the pathogen’s respiratory chain by binding to the ubiquinol oxidation center (Qo) site of the cytochrome bc1 enzyme complex (complex III)^8^, have activity against virtually all oomycetes and fungal phytopathogens^9^. However, because QoIs affect a specific, single site, resistance to QoIs has rapidly developed in many pathogens. QoI resistance is largely due to a single amino acid substitution in the target cytochrome b protein (CYTB) of the pathogen. The main substitutions or changes are: glycine (G) to alanine (A) at position 143 (G143A), phenylalanine (F) to leucine (L) at position 129 (F129L), and glycine (G) to arginine (R) at position 137 (G137R)^8^,^10^. In most cases, the G143A mutation leads to high resistance^11^,^12^, while the F129L and the G137R mutations lead to moderate resistance^9^,^13^,^14^. Esser L *et al.*^15^ used crystallographic data of CYTB from bovin heart that was bound with different QoI inhibitors to explain how mutations in CYTB can reduce binding of QoI inhibitors and thereby result in resistance. In spite of this, how the shape of the QoI fungicide molecules conforms to the binding site on the mutants and how the point mutations affect fungicide binding are incompletely understood.

Enestroburin^16^,^17^,^18^,^19^, SYP-1620^20^,^21^, SYP-2815, and ZJ0712^22^,^23^ are novel QoI fungicides have been recently developed in China (Fig. 1). For those novel QoI fungicides, mutants with high or moderate resistance have been obtained in the laboratory for the following combinations of QoI and pathogen: enestroburin and *Pseudoperonospora cubensis*^19^, enestroburin and *Magnaporthe grisea*^24^, SYP-1620 and *M. grisea*^25^, and ZJ0712 and *Sphaerotheca fuliginea*^22^. In our preliminary work, eight mutants of *P. litchii* with different resistance level resistant to three novel QoI fungicides (SYP-1620, SYP-2815, and ZJ0712) and azoxystrobin (as a known QoI fungicide) were obtained by exposing field isolates to fungicides or UV light in the laboratory^26^. The objectives of this study were to: (1) compare the point mutations in the CYTB of *P. litchii* that result in different levels of resistance to the fungicides; (2) investigate how the point mutations might affect QoI fungicide binding by constructing docking models.

## Results

### Detection of point mutations on the hot spot region of the *cytb* genes

**Figure 2:**
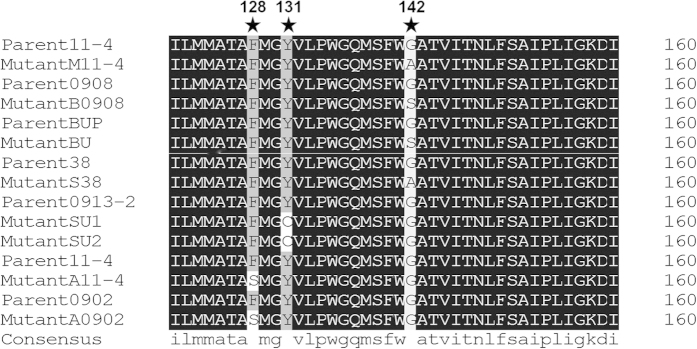
Site of mutation in the CYTB protein associated with QoI fungicide resistance. Alignment of partial amino acid sequences of CYTB protein from QoI fungicide-resistant mutants and their parents of *Peronophythora litchii*. Mutations in QoI-resistant mutants are indicated by asterisks.

Primers PlcytbF2/ PlcytbR2, which were designed to amplify the *cytb* gene in *P. litchii*, produced a single bright band of 935 bp with both gDNA and cDNA (wild-type isolate 38). This fragment included possible mutations that can confer resistance to QoI fungicides (amino acid residues 120–160 and 250–300)^27^. The same primers were also used for the QoI fungicide-resistant mutants and their parents that are listed in Table 1, and they all yielded the same amplification products of 935 bp. The sequencing of these products revealed several point mutations in the QoI fungicide-resistant mutants (Fig. 2) (Table 2). In mutants M11-4 and S38, the amino acid substitution of glycine with alanine was detected at position 142 (G142A) of the CYTB protein. In mutants B0908 and BU, the substitution of glycine with serine was detected at the same position (G142S). A second target-site mutation, the substitution of phenylalanine with serine (F128S), was detected at position 128 in mutants A11-4 and A0902. The substitution of tyrosine with cysteine (Y131C) was detected at position 131 in mutants SU1 and SU2.

### Amplification and analysis of the *cytb* gene sequence

Inverse PCR with three pairs of nested primers (GSP1/GSP5, GSP2/GSP5, and GSP7/GSP8) (Table 3) was carried out for *P. litchii* (wild-type isolate 38) to amplify the cDNA 5′ end of the *cytb* gene; a 500-bp fragment was obtained. The sequencing results showed that this fragment included the 5′ end of the *cytb* gene in *P. litchii*. The 3′ RACE system with the designed nested primers (Table 3) was used for rapid amplification of the cDNA 3′ end of the *cytb* gene. A 250-bp band was cloned, which included the sequence information of the cDNA 3′ end of the *cytb* gene. The full length of the *cytb* gene in *P. litchii* was 1,149 bp, which encoded 382 amino acids.

### Binding environment of the five QoI fungicides in the wild-type Qo-binding pocket of bovine heart

**Figure 3:**
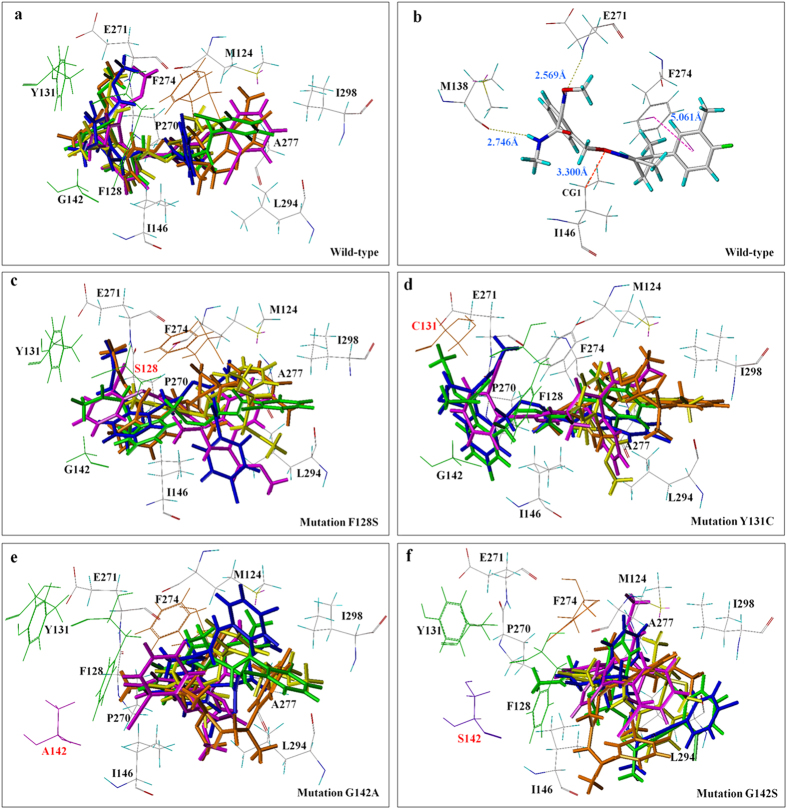
3D representation of QoI fungicides in the docking pocket of Qo with mutations and without mutations. (**a**) Superposition of QoI fungicides in the docking pocket of wild-type Qo. The fungicides (and color codes) are: azoxystrobin (magenta), SYP-1620 (green), ZJ0712 (yellow), SYP-2815 (orange), and enestroburin (blue). (**b**) Docking interactions of SYP-2815 with selected amino acid residues at the wild-type Qo-docking pocket. Hydrogen bond is indicated with yellow dashes; van der Waals interaction is indicated with red dashes; π-π stacking is indicated with magenta dashes. (**c–f**) Superposition of QoI fungicides in the docking pockets of Qo with the following mutations: F128S, Y131C, G142A, and G142S were poor compared with those in the wild-type docking pocket (**a**). (**e,f**) show fungicide molecules were obviously kept away from the amino acid A or S at position 142. The fungicides (and color codes) are: azoxystrobin (magenta), SYP-1620 (green), ZJ0712 (yellow), SYP-2815 (orange), and enestroburin (blue).

The binding conformation of azoxystrobin to bovine heart CYTBC1 derived from the docking result revealed a similar conformation to that in crystal structure “1SQB”^15^. The root mean square deviation (RMSD) between the docked conformation and the crystal one was 0.67 with a docking score of 11.75 (Table 4). It is believed that the docking method should be useful for studying the binding conformation of the four novel QoI fungicides to the target (Fig. 3a). The methoxy methyl acrylate groups of the four novel QoI fungicides inserted between the residues F128 and Y131 of the C-helix, and hydrogen bonds, π-π stacking and van der Waals interaction existed in the binding of the four novel QoI fungicides to the wild-type Qo-binding pocket (Fig. 3b). The docking scores for the four QoI fungicides were shown in table 4. SYP-2815 was the second highest one after azoxystrobin, suggesting a relatively strong binding affinity relative to SYP-1620, ZJ0712, and enestroburin.

### Analysis of the affinity between the mutation positions and the QoI fungicides

**Figure 4:**
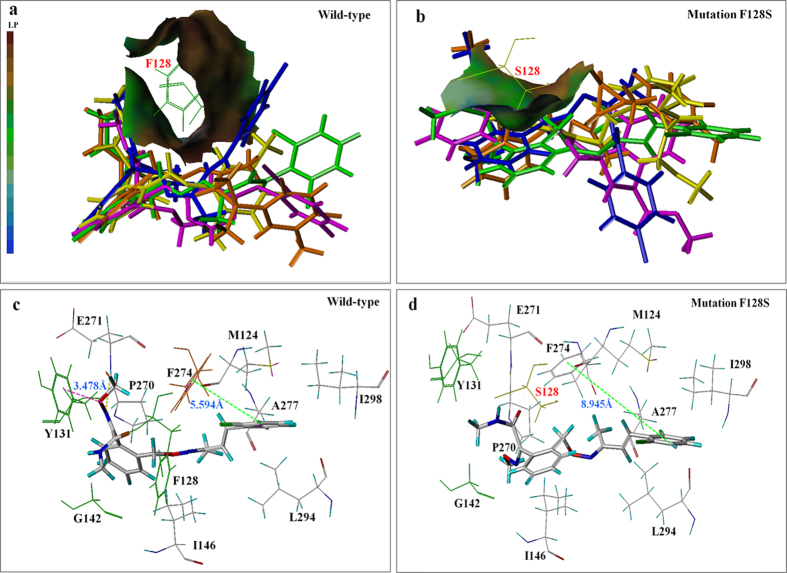
Docking interactions of the wild-type Qo pocket and the mutant type (F128S) Qo pocket with QoI fungicides. (**a**) Indicates QoI fungicides are bond to a highly hydrophobic surface of Qo pocket including F128 (brown molecular surface). LP indicates the lipophilic potential; as the color of the molecular surface becomes browner, the surface is more lipophilic and more hydrophobic, but as the color becomes bluer, the surface becomes more hydrophilic and less hydrophobic. The fungicides (and color codes) are: azoxystrobin (magenta), SYP-1620 (green), ZJ0712 (yellow), SYP-2815 (orange), and enestroburin (blue). (**b**) Indicates QoI fungicides are bond to a less hydrophobic surface of Qo pocket (less brown molecular surface) when F is substituted by S at position 128. (**c**) 3D representation of SYP-1620 with the amino acid residues forming the wild-type Qo-binding pocket, and hydrogen bond (yellow dashes), π-interaction (magenta dashes) and π-π stacking (green dashes) were shown. (**d**) 3D representation of SYP-1620 with the amino acid residues forming Qo-binding pocket with the F128S mutation, π-π stacking almost disappeared when the distance between the side-chain of fungicides and the aromatic ring of F274 increased to 8.945 Å (green dashes).

Models were built of the five QoI fungicides docking into the Qo-binding pocket with the following mutations: F128S, Y131C, G142A, and G142S (Fig. 3c–f). The decreased docking scores of the predicted models were showed in Table 4. The model indicates for the mutation F128S that the changed conformation of the Qo-binding pocket reduces the binding of the five QoI fungicides, and that the superposition of QoI fungicides in the docking pocket was poor (Fig. 3c). The mutation F128S reduces hydrophobic interactions between the methyl group of the QoI fungicides and the aromatic ring of F128 and thereby weakens the binding of the fungicides to the Qo-binding pocket (Fig. 4b). This mutation also weakens hydrogen bonds, π-π stacking, and van der Waals interaction. Especially for SYP-1620 (Fig. 4d) and enestroburin, π-π stacking almost disappeared when the distance between the side-chain of fungicides and the aromatic ring of F274 increased to 8.945 Å (Fig. 4d) and 8.806 Å, while the interaction was unaffected for azoxystrobin (4.192 Å) and ZJ0712 (4.387 Å). In the case of SYP-2815, π-π stacking was weaker but there was a strong hydrogen bond (1.840 Å). This may explain why the mutation F128S (mutants A11-4 and A0902) resulted in high resistance to SYP-1620 (RF > 500) and enestroburin (RF > 100) but in only low or moderate resistance to the other three novel fungicides (Table 1).

The mutation Y131C also changed the binding of the fungicide with the Qo site (Fig. 3d). In the wild type, the methoxy methyl acrylate groups of the four novel QoI fungicides insert between the residues F128 and Y131, creating a π interaction (Fig. 4c) between the aromatic ring of Y131 and the oxygen atom of the active group, and the distances were all less than 5 Å. When Y131 is replaced by C, however, the π interaction and hydrogen bond were missing. π-π stacking became weak or absent. The distance between the oxygen atom of the active group and CG1 of Ile146, which could provide a van der Waals interaction, became large and weakened the combination. These effects could explain why the mutation Y131C results in high resistance to QoI fungicides (RF > 100).

**Figure 5:**
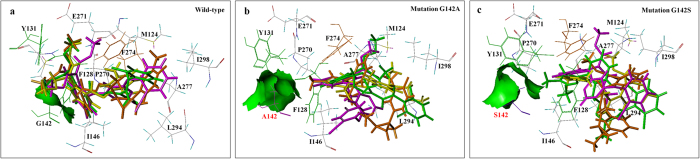
Molecule surface of QoI fungicide interactions with the amino acid at position 142 from wild-type Qo pocket and the mutant type (G142A and G142S) pockets. (**a**) The van der Waals surface (shown in green surface) of the fungicide and the amino acid G142 of the wild type were sunken, indicating that the steric hindrance was small. (**b,c**) show that green van der Waals surface was raised, indicating an increase in steric hindrance with the mutations G142A and G142S. The fungicides (and color codes) are: azoxystrobin (magenta), SYP-1620 (green), ZJ0712 (yellow), and SYP-2815 (orange).

As was the case with the other mutations, the mutation G142A/G142S alters the binding site for the fungicides, and fungicide molecules were obviously kept away from the amino acid at position 142 (Fig. 3e,f). In this case, the hydrogen bond is missing, π-π stacking becomes weak or even missing, and the van Der Waals interaction becomes weak and weakens the binding. According to analysis of the molecular surfaces (Fig. 5), the van der Waals surface of the fungicide and the binding site of the wild type were sunken, indicating that the steric hindrance was small. When this site was mutated to A or S, however, the van der Waals surface was raised, indicating an increase in steric hindrance; this would weaken the binding and result in high resistance to QoI fungicides (RF > 100).

## Discussion

QoI fungicides are generally considered to have a high resistance risk in many plant pathogen species such as downy mildews, anthracnose^8^,^9^,^13^. However, no QoI fungicides resistance in *P. litchii* has been documented till now. In our study, *P*. *litchii* mutants with high levels of resistance to SYP-2815, ZJ0712, and SYP-1620, as well as to the reference fungicide azoxystrobin, were obtained in the laboratory by exposing isolates to increasing fungicide concentrations on agar, which might indicate high resistance risk development of *P. litchii* to QoI fungicides in real fields.

The nucleotide sequences of the mitochondrial *cytb* gene were compared between the mutants and the sensitive wild type, and three single-site amino acid substitutions (G142A/S, Y131C, and F128S) were found on the CYTB protein of different mutants with high resistance to the novel QoI fungicides. The G142S or G142A substitution was equivalent to the G143S substitution that was previously associated with high resistance of *M. grisea* to azoxystrobin^28^. The G143A substitution has been identified as the most common cause of QoI resistance and has been detected in *Blumeria graminis* f. sp. *tritici*, *B. graminis* f. sp. *hordei*, *S. fuliginea*, *P. cubensis*, *Plasmopara viticola*, and *Mycosphaerella fijiensis*^27^,^29^. The G142S or G142A substitution in *P*. *litchii* was associated with a high level of resistance (RF > 100) to all the tested QoI fungicides. Our results once again demonstrate that substitution of the amino acid at this position is closely associated with the failure of QoIs to control disease. The second mutation detected in the current study, Y131C, leads to high resistance to all QoI fungicides (RF > 100), but it was obtained with UV radiation and has not been detected in nature. The third mutation, F128S, is similar to the F129L mutation that often confers moderate (partial) resistance to QoI fungicides in *Pyricularia grisea*^29^ and *Pythium aphanidermatum*^9^. In our study, the F128S substitution in *P*. *litchii* was associated with a high level of resistance to SYP-1620 (RF > 500) and enestroburin (RF > 100) and with lower levels of resistance to SYP-2815, ZJ0712, and azoxystrobin.

A drug molecule will be most potent when its bioactive conformation matches that of its target’s binding pocket^30^. When the conformation of the binding pocket changes, however, the drug may become less effective, i.e., resistance may develop. We investigated the relationships between the molecular mechanism of QoI fungicide-resistance and the stereochemistry of the fungicide and the Qo-binding site. *Saccharomyces cerevisiae* was previously used as a model system to characterize the relationship between CYTB mutations in *P. megasperma* and the pathogen’s resistance to Qo inhibitors^31^. Like several previous studies of fungicide resistance and fungicide development^32^,^33^, we used a complex crystal structure of CYTBC1 from bovine heart mitochondria bound with a QoI fungicide named azoxystrobin to study the molecular docking of novel QoI fungicides to the target site.

The docking score for SYP-2815, ZJ0712, and enestroburin in the wild-type CYTBC1 protein was consistent with the biological activity data of the three novel QoI fungicides (Table 4). However, there is a more flexible conformation in SYP-1620, which makes it match the Qo-binding pocket well with a higher docking score than ZJ0712 and enestroburin (Table 4). Although the scores of the predicted model are only partly in line with the respective mean EC_50_ values for the fungicides (Table 4), the models we constructed are useful for describing and comparing how the QoI fungicides form complexes with mutated and wild-type binding pockets. The results indicated that the steric hindrance caused by the amino acid mutation at position 142 (G142A/S) is likely to abolish the binding of this class of Qo antagonists, which confirms an earlier report^31^.

Previous research indicated that resistance to stigmatellin in yeast with the F129L mutation could be due to a subtle alteration of the backbone fold at Qo that reduced the antagonist’s access to the Qo site, but that limited cross-resistance to azoxystrobin and sensitivity to pyraclostrobin was likely due to the difference in pharmacophore structure between these two compounds^31^. Our models on the effect of F128S mutation on the binding of fungicides to Qo indicated that the mutation reduced hydrophobic interactions and other interactions and that the effects of the F128S mutation differed for the five QoI fungicides. π-π stacking almost disappeared for SYP-1620 and enestroburin, remained for azoxystrobin and ZJ0712, and was reduced for SYP-2815. This is consistent with the bioassay results, which indicated that the F128S mutation resulted in high resistance to SYP-1620 and enestroburin and low or intermediate resistance to the other three fungicides. We also provided insight into how the Y131C mutation could alter the Qo-binding pocket and therefore result in high resistance to QoI fungicides, although this mutation was generated by exposure to UV irradiation.

In conclusion, it is the first time to show a comparative study of resistance mechanisms of *P. litchii* to four novel QoI fungicides developed in China and use molecular docking to predict how the mutations alter the binding of QoI fungicides to the Qo-binding pockets of *P. litchii*, which increased our understanding of QoI fungicide-resistance mechanisms and may help in the development of more potent inhibitors to manage plant diseases in the fields.

## Methods

### Isolates of *P. litchii*

In our preliminary work, eight mutants of *P. litchii* with different reistance level resistant to three novel QoI fungicides (SYP-1620, SYP-2815 and ZJ0712) and azoxystrobin (as a known QoI fungicide) were obtained by exposing field isolates to fungicides or UV light in the laboratory, including one ZJ0712-resistant mutant (BU) was obtained from parent BUP and two SYP-2815-resistant mutants (SU1 and SU2) were obtained from parent 0913-2 by exposing parent isolates to UV radiation in the laboratory; one SYP-2815-resistant mutant (S38) was obtained from parent 38, two SYP-1620-resistant mutants (A11-4 and A0902) were obtained from parent 11-4, one ZJ0712-resistant mutant (B0908) was obtained from parent 0908, and one azoxystrobin-resistant mutant (M11-4) was obtained from parent 11-4 by exposing parent isolates to fungicides in the laboratory. Mutants resistant to enestroburin were not obtained. All eight mutants showed a high and stable resistance relative to parental isolates (Table 1), and the fitness of the all mutants were similar to or better than that of the parental isolates^26^. Interestingly, the resistance of mutants A11-4 and A0902, which were selected on media containing SYP-1620, was high against SYP–1620 and enestroburin but was much lower against SYP-2815, ZJ0712, and azoxystrobin (Table 1)^26^.

### Genomic DNA and RNA extraction

The QoI fungicide-resistant mutants and their parents of *P. litchii* (Table 1) were grown on cellophane on white kidney bean agar (WKBA) (60.0 g of boiled white kidney beans powder and 12.5 g of agar, with deionized H_2_O brought to 1 liter) at 25 °C in the dark for 6 days before the mycelium was harvested. Genomic DNA (gDNA) was extracted with the CTAB procedure^34^, and total RNA was extracted with the SV Total RNA Isolation System (Promega Corp., Beijing, China). RT-PCR was carried out with cDNA Synthesis Kits (Life Technologies Corp., Beijing, China).

### Detection of point mutations on the hot spot region of the *cytb* genes

Oligonucleotide primers (PlcytbF1 and PlcytbR1, Table 3), designed based on the conserved sequence of the apocytochrome b genes or the cytochrome b gene in *Phytophthora infestans* (AAF24777.1), *Phytophthora sojae* (ABG54046.1), *Phytophthora ramorum* (ABG54094.1), and *Phytophthora megasperma* (AAA32026.2) in the GenBank database, were used to amplify the partial cytochrome b (*cytb*) gene fragment (~607 bp) from gDNA in *P. litchii* (wild-type isolate 38). The primers PlcytbF2 and PlcytbR2 (Table 3), which were also designed based on the same sequences, were used to amplify a longer *cytb* gene fragment from gDNA and cDNA (wild-type isolate 38). All primers were synthesized by Beijing Sunbiotech Co. Ltd. (Beijing, China). Polymerase chain reaction (PCR) was performed in a total reaction volume of 25 μl containing 2.5 μl of 10× *Taq* reaction buffer (2.5 μM, TransGen Biotech, Beijing, China), 1 μl of dNTP (10 mM each, TransGen Biotech, Beijing, China), 1 μl of each primer (10 μM), 10 ng of DNA template, 18 μl of H_2_O, and 2.5 U of *Taq* DNA polymerase (TransGen Biotech, Beijing, China). Cycling parameters were 95 °C for 5 min; followed by 35 cycles of 95 °C for 30 s, 60 °C for 30 s, and 72 °C for 1 min; a final extension of 10 min at 72 °C; and then cooling to 4 °C. All products of the expected size were separated and purified by electrophoresis in a 1% agarose gel in Tris-acetate (TAE) buffer and were cloned into the pEASY-T3 Vector (TransGen Biotech, Beijing, China) and sequenced by Beijing Sunbiotech Co. Ltd. (Beijing, China). The programs in the DNAMAN software were used to predict the CYTB amino acid sequences in *P. litchii* and to compare the CYTB amino acid sequences of the wild-type isolates with those of the QoI-resistant mutants.

### Amplification and analysis of the *cyt b* gene flanking sequence of *P. litchii*

Inverse polymerase chain reaction (IPCR) and 3′ rapid amplification of cDNA ends (3′ RACE) were used to amplify the flanking fragment of *cytb* in *P. litchii*. IPCR was performed according to the procedure previously reported^35^, with slight modifications. Briefly, 2 μl (300 ng/μl) of gDNA of *P. litchii* (wild-type isolate 38) was digested with 1 μl of *Eco*R I (Thermo Scitific, Shanghai, China) in a total volume of 20 μl at 37 °C for overnight; the enzyme activity was inactivated by heating at 65 °C for 20 min. Circularization was conducted in a total reaction volume of 200 μl containing 4 μl of digesting DNA products, 5 μl of T4 ligase (TransGen Biotech, Beijing, China), 20 μl of 10× T4 DNA ligase buffer (TransGen Biotech, Beijing, China), and 171 μl of ddH_2_O. After incubation at 25 °C for 10 min, the circularized DNA was purified with the CTAB procedure^34^. The circularized DNA was amplified using gene-specific primers (GSP, Table 3) designed according to the partial *cyt b* gene sequence and targeted toward the flanking sequences. All primers were synthesized by Beijing Sunbiotech Co. Ltd. (Beijing, China). The first round of IPCR amplifications was performed in a total reaction volume of 50 μl containing 5 μl of 10× *Taq* reaction buffer (2.5 μM, TransGen Biotech, Beijing, China), 2 μl of dNTP (10 mM each, TransGen Biotech, Beijing, China), 2.5 μM of each primer (10 μM, GSP1 and GSP5), 5 ng of circularized DNA template, 32 μl of H_2_O, and 5 U of *Taq* DNA polymerase (TransGen Biotech, Beijing, China). Cycling parameters were 94 °C for 5 min; followed by 35 cycles of 94 °C for 30 s, 62 °C for 30 s, and 72 °C for 3 min; and a final extension of 10 min at 72 °C. The first IPCR products, diluted 10-fold and 100-fold, were used as template for the second round of IPCR. The same reaction system was used as for the first amplification except that the primers were replaced with two pairs of nested primers: GSP2/GSP5 and GSP7/GSP8. Cycling parameters were as described for the first round except that both 60 °C and 64 °C were used as the annealing temperature.

The 3′ RACE system (Life Technologies Corp., Beijing, China) was carried out according the manufacturer’s protocol to obtain the 3′ end of the *cytb* gene in *P. litchii* (wild-type isolate 38). Nested amplification was performed, and primers GSP8 and GSP14 were used in the first round of PCR. Cycling parameters were 95 °C for 5 min; followed by 30 cycles of 95 °C for 30 s, 52 °C for 30 s, and 72 °C for 2 min; and a final extension of 10 min at 72 °C. The two pairs of primers GSP8/AUAP and GSP10/AUAP were used for the second round of PCR, with previous amplification products diluted 1000-fold as the template. The same reaction system and program were used as in the first round of PCR. Products of the expected size were separated, purified, sequenced, and analyzed as described above.

### Computational methods of molecular docking

For docking studies of QoI fungicides in the Qo-binding pocket, the crystal structure of mitochondrial cytochrome bc1 enzyme complex (CYTBC1) from bovine heart bound with azoxystrobin was retrieved from the Protein Data Bank (PDB entry 1SQB). Sequence alignment of the CYTB subunit by the DNAMAN software indicated that the CYTB amino acid residues of bovine heart are similar to those of *P. litchii* with 83.07% sequence identity. Therefore, the crystal structure 1SQB was used as the template to study the binding conformation of four novel QoI fungicides in the Qo binding pocket. The 3D conformations of five QoI fungicides were optimized using MMFF94 force field^36^ with MMFF94 charge by the Sybyl 7.3 software package on the Linux platform. Docking experiments were carried out using the Surflex-Dock algorithm of the Sybyl 7.3 software. The best ligand pose was selected based on the top Surflex-Dock energy score^37^. Then the Qo binding pocket from “1SQB” was site-directed mutated at the detected residues including F128S, Y131C, G142A, and G142S with PyMOL software, respectively, and was minimized in energy using the MMFF94 force field. Five QoI fungicides were docked into the mutated Qo binding sites. The relationships between the mutation sites and the affinity of fungicides were analyzed based on the energy score and binding mode to the Qo binding site.

## Additional Information

**How to cite this article**: Zhou, Y. *et al.* Resistance Mechanisms and Molecular Docking Studies of Four Novel QoI Fungicides in *Peronophythora litchii*. *Sci. Rep.* **5**, 17466; doi: 10.1038/srep17466 (2015).

## Figures and Tables

**Figure 1:**
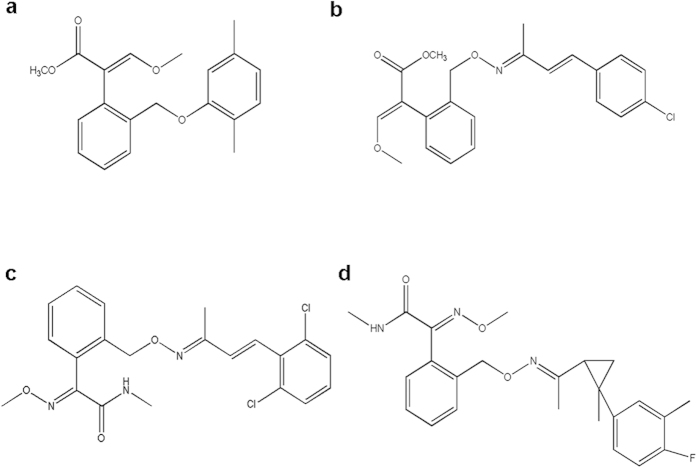
Chemical structures of four QoI fungicides. (**a**) ZJ0712. (**b**) enestroburin. (**c**) SYP-1620. (**d**) SYP-2815.

**Table 1:** Sensitivity of eight QoI fungicide-resistant mutants of *Peronophythora litchii* to four QoI fungicides.

Isolate		Origin^a^	RF^b^				
Parent	Mutant		Azoxystrobin	SYP-2815	ZJ0712	SYP-1620	Enestroburin
11–4	M11–4	Azoxystrobin in media	>9000	>500	>500	>200	>100
38	S38	SYP-2815 in media	>4000	>2000	>500	>500	>100
0913-2	SU1	UV radiation	489	294	>600	>191	>100
0913-2	SU2	UV radiation	225	184	190	>191	>100
0908	B0908	ZJ0712 in media	>2500	>500	>1000	>500	>100
BUP	BU	UV radiation	>5000	>500	>1000	>500	>100
11–4	A11–4	SYP-1620 in media	14.32	29.16	3.02	>1000	>100
0902	A0902	SYP-1620 in media	15.56	58.15	9.84	>500	>100

^a^Mutants were obtained by exposure of field isolates (parents) to fungicide in media or to UV light.

^b^RF, Resistance factor, a ratio of EC_50_ for a fungicide-resistant mutant relative to EC_50_ for the parental isolate.

**Table 2:** Differences in nucleotide and amino acid sequences between *Peronophythora litchii* QoI fungicide-resistant mutants and their QoI fungicide-sensitive parents.

Parental isolate and mutants*	Nucleotide at the indicated position on the cytochrome b gene				Amino acid at the indicated position on the CYTB protein		
	383	392	424	425	128	131	142
11-4	T	A	G	G	F	Y	G
M11-4	T	A	G	**C**	F	Y	**A**
B0908	T	A	**A**	G	F	Y	**S**
BU	T	A	**A**	G	F	Y	**S**
S38	T	A	G	**C**	F	Y	**A**
SU1	T	**G**	G	G	F	**C**	G
SU2	T	**G**	G	G	F	**C**	G
A11-4	**C**	A	G	G	**S**	Y	G
A0902	**C**	A	G	G	**S**	Y	G

^*^11-4 is a parent isolate (QoI fungicide-sensitive), all others are QoI fungicide-resistant mutants. Compared with the parent isolate 11-4, the substitution nucleotide or amino acid for specific mutants are presented in bold font.

**Table 3:** Primers used for the amplification of the partial cytochrome b gene sequence of *Peronophythora litchii*.

Primer	Sequence (5′-3′)
PlcytbF2	GGATTTGGTTCGTTAGCCGGT
PlcytbR2	CATTGTCCAACCCAACCTAAT
GSP1	CAGCAAAACCTCCCCATAACCAATCAAC
GSP2	ACAGTTGCACCCCAAAAACTCATTTGTC
GSP5	GGAGGTGTTATTGCAATGTTTGGTTCG
GSP7	AATTACACCTGAGCACCATAAAGC
GSP8	TAGAAGTACCGCTTTTAGACC
GSP10	ATTAGGTTGGGTTGGACAATG
GSP14	GTTTTACCTTGGGGACAAATGAG

**Table 4:** Docking scores of five QoI fungicides with the Qo binding site in wild-type and site-directed mutagenesis bovine heart.

Fungicide	Mean EC_50_(μg/ml)*	Docking scores				
		Wild-type	S128	C131	A142	S142
Azoxystrobin	0.1228	11.75	7.93	5.67	6.14	6.59
SYP-2815	0.1385	8.95	5.56	4.10	4.71	5.36
SYP-1620	2.7234	7.85	4.87	4.69	4.70	4.35
ZJ0712	0.1478	5.99	5.28	2.23	4.68	4.10
Enestroburin	0.8103	5.96	5.23	4.98	4.78	3.89

^*^EC_50_ = effective concentration for 50% inhibition of mycelial growth.
